# Angiolymphoid hyperplasia with eosinophilia: excellent response to intralesional triamcinolone

**DOI:** 10.1016/S1808-8694(15)30771-0

**Published:** 2015-10-19

**Authors:** Roberto Rheingantz da Cunha Filho, Hiram Larangeira de Almeida

**Affiliations:** 1 M. Sc. In Health and Behavior, Dermatologist – CEM – Centro de Especialidades Médicas de Joaçaba (SC); 2 PhD in Dermatology, Professor of Dermatology UFPel e UCPel, Professor and Coordinator of the Graduate Course in Health and Behavior – UCPel (RS)

**Keywords:** triamcinolone, angiolymphoid hyperplasia with eosinophilia, ear, ear diseases, external

## CLINICAL CASE PRESENTATION

**Figure 1:**
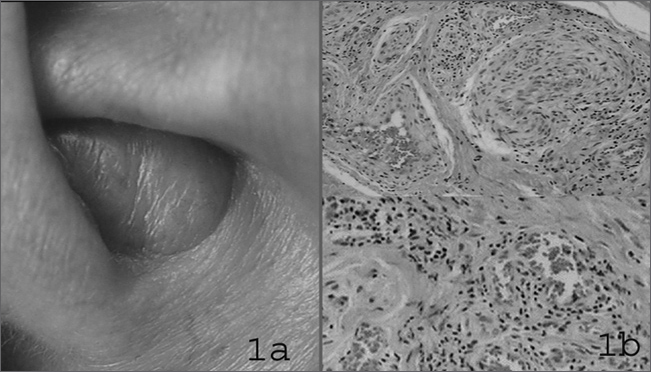
Angiolymphoid hyperplasia with eosinophilia. 1a – clinical close up of the ear lesion. 1b – histology, prevailing vascular alteration with histiocytic epithelium, some lymphoid-type follicles and a few eosinophils.

A 45 year-old white housewife, came with a slightly purple and painless nodular lesion that had been growing for 4 years, bleeding spontaneously and sporadically, on her left ear (Figure 1a). The lesion was surgically excised, but relapsed promptly. CBC, platelets, total IgE, prothrombin time test, activated partial thromboplastin time and urine exam were normal. No history of atopy or chronic diseases.

Pathology exam showed a benign vascular proliferation, with enlarged endothelial cells and histiocytic appearance, inflammatory infiltrate with lymphocytes and some eosinophils and rare lymphoid follicular formations (Figure 1b). We injected 0.5ml of 20mg/2ml triamcinolone solution in the lesion. In a few days there was lesion necrosis with ulceration, followed by re-epithelization and complete resolution at 90 days of follow up.

## DISCUSSION

There is certain confusion between Kimura's Disease and angiolymphoid hyperplasia with eosinophilia; however, they represent different entities, with different clinical manifestations and behaviors, despite their histopathological similarities. It is possible to differentiate them clinically and histopathologically.^2^^,^^3^

In general, the former takes on a more systemic and tumoral characteristic, preferably affecting Asian patients. It is possible to find an increase in total IgE and eosinophils in their serum. Pathology shows inflammatory cells forming large clusters, like lymphoid follicles with germinative centers, large number of eosinophils and mild vascular alterations.^2^^,^^3^^,^^4^

The latter, which is the present case, manifests a lonely nodule in the subcutaneous or the mucosa, usually on the head and neck. It can be asymptomatic or present with pruritus. It is rarely found on other parts of the body, such as shoulders, penis, lungs, nail beds and adjacent phalanx. It has been reported associations with nephrotic syndromes or kidney diseases, squamous cell carcinoma and arteriovenous malformations. These associations are influenced by chance, sometimes making it difficult to explain the cause, thus the patient requires further investigation. Histology shows vascular alterations, with proliferated endothelial cells, large in size and with histiocytic appearance, chronic infiltrate made up of lymphocytes, with varied number of eosinophils, which can appear in lesser number. Lymphoid follicles are few or inexistent.^2^^,^^4^

Differential diagnosis to consider: cavernous hemangioma, granuloma pyogenic, facial granuloma, Kaposi sarcoma, angiomatous lymphoid hamartoma, periarteritis nodosa, pseudolymphoma.^2^

There are different treatment modalities, such as conventional surgery, Mohs's procedure, laser, steroids, alpha 2-a interferon, intralesional chemotherapy, calcineurin inhibitors and imiquimod, cryotherapy and radiotherapy, with variable results, but usually positive.^5^ The major mechanisms through which triamcinolone acts on the lesion must be: angiogenesis inhibition, lymphocytes and eosinophils apoptosis. Other effects that may impact the cure are vasoconstriction, reduction in vascular permeability, transcription (cytokines) maturation, proliferation and activity factors inhibition on inflammatory cells. Since it is a benign condition, aggressive approaches such as surgery or aggressive medication must not be attempted.

## FINAL REMARKS

Most of this literature is present in dermatology journals; however, professionals who work with diseases that may involve the head and neck should also be attentive and consider the diagnosis of angiolymphoid hyperplasia with eosinophilia in nodular lesions, such as Kimura disease in tumoral lesions. Histopathology is obviously essential. Intra-lesion steroid injection is a viable treatment option.
